# Unmasking Evans Syndrome: A Case of New Onset Anemia and Thrombocytopenia in a Pregnant Patient With Systemic Lupus Erythematosus

**DOI:** 10.1155/crh/9110168

**Published:** 2026-07-02

**Authors:** Christian Bittner, Niels A. Ryden, Charles Jacocks, Chung-Ting J. Kou

**Affiliations:** ^1^ Department of Internal Medicine, Brooke Army Medical Center, San Antonio, Texas, USA, bamc.amedd.army.mil; ^2^ Department of Hematology & Oncology, Brooke Army Medical Center, San Antonio, Texas, USA, bamc.amedd.army.mil

## Abstract

Evans Syndrome (ES) is an immune‐mediated disorder defined by the presence of two or more autoimmune cytopenias, typically autoimmune hemolytic anemia (AIHA) and immune thrombocytopenic purpura (ITP), with or without associated neutropenia. The disease can be classified as primary (i.e., idiopathic) or secondary to other underlying conditions, such as systemic lupus erythematosus (SLE), lymphoproliferative disorders, infections, and immunodeficiency states. We present a case of ES in a 21‐year‐old pregnant woman (G1P0, 19 weeks and 2 days gestation) secondary to SLE. The patient was treated with high‐dose steroids and intravenous immunoglobulin therapy (IVIG), resulting in marked improvement in her cell counts. Following delivery, she was diagnosed with SLE and began treatment with rituximab and hydroxychloroquine, leading to remission of her symptoms and normalization of her anemia and thrombocytopenia. The varied presentations and associated conditions make ES a challenging diagnostic conundrum. Differentiating between primary and secondary ES is crucial for prognosis and management.

## 1. Introduction

Dr. Robert Evans first described Evans Syndrome (ES) in 1951 as the concomitant occurrence of immune thrombocytopenic purpura (ITP) and autoimmune hemolytic anemia (AIHA) [1]. It is now defined by the presence of at least two autoimmune cytopenias, most commonly with AIHA and ITP with or without neutropenia. ES accounts for 0.3%–7% of AIHA cases and 2%–2.7% of ITP cases [2, 3]. The type of hemolytic anemia that presents in ES is a warm AIHA with IgG antibodies reacting to erythrocyte surface antigens at regular body temperatures, which leads to extravascular hemolysis and the destruction of red blood cells in the spleen. ITP, on the other hand, involves the immune system targeting GPIIb/IIIa and GPIb/IX on the patient’s platelets marking them for destruction by macrophages in the spleen and liver via Fcγ receptor‐mediated phagocytosis. Neutropenia has also been documented, and these cytopenias can present together at the time of diagnosis or sequentially. The condition is the result of B lymphocytes producing autoantibodies that attack the patient’s own cells; however, the exact cause of ES is currently unknown. Despite a slight female predominance (51%–60%), there are very few reported cases of ES occurring in pregnancy [4]. In this case report, we describe a rare presentation of ES occurring in a young pregnant woman seen by our hematology department.

## 2. Case Report

### 2.1. Investigations

A 21‐year‐old pregnant woman (G1P0, 19 weeks and 2 days gestation) with minimal past medical history presented to the emergency department with one week of malaise. She had decreased oral intake, dehydration, headache, nausea, weakness, and fatigue but denied associated mucocutaneous bleeding, petechiae, melena, hematochezia, gross hematuria, or vaginal bleeding. She did not have any other relevant family and social history. A clinical timeline is listed below in Table 1 to aid in visualizing overall clinical course.

**TABLE 1:** Clinical timeline of patient’s case.

Clinical timeline			
Timepoint	Clinical events	Key laboratory findings	Management
19w2d gestation (Admission #1)	Malaise, fatigue, nausea, weakness	Hgb 7.0 g/dL; Plt 50,000/µL; ferritin 5181 ng/mL; haptoglobin < 10 mg/dL; schistocytes; DAT negative; ADAMTS13 77.2%; AST 55 U/L	Admitted; 3 days plasma exchange (PLEX)
Discharge (Admission #1)	Clinical improvement	Plt 57,000/µL; Hgb 8.0 g/dL	No outpatient medications
29 weeks gestation (Admission #2)	Recurrent severe anemia and thrombocytopenia	Hgb 8.4 g/dL; Plt 6000/µL; Spherocytes; DAT positive (IgG); No schistocytes	High‐dose steroids + IVIG; discharged on taper
Delivery	Uncomplicated	—	Continued steroid taper
1–4 months postpartum	Persistent thrombocytopenia& new arthralgias	Positive ANA, anti‐dsDNA, anti‐SSA/Ro, anti‐Smith	Diagnosed SLE
4 months postpartum	Final diagnosis: ES secondary to SLE	—	Rituximab + hydroxychloroquine initiated
14 months follow‐up	Resolution of arthralgias	Hgb 10.7 g/dL; Plt 403,000/µL	Sustained remission

### 2.2. Diagnosis

**FIGURE 1:**
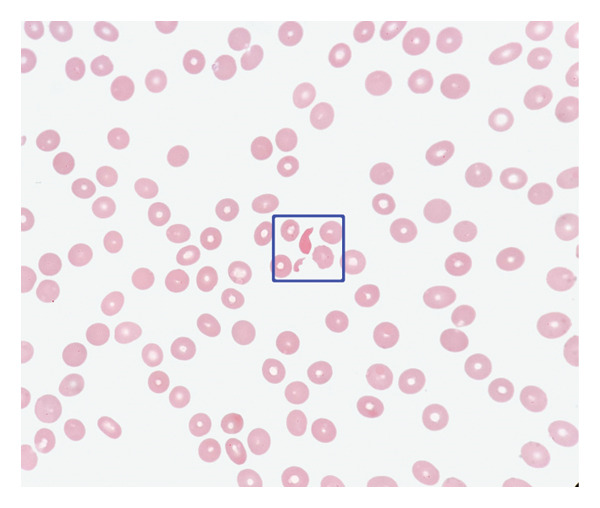
Peripheral blood smear with a pair of schistocytes visible in the center of the image.

Physical examination was unremarkable aside from mild tachycardia. Her initial labs showed anemia and thrombocytopenia, and she was advised to return to the OBGYN clinic in 2 days for iron infusions due to suspected iron deficiency anemia. However, upon her arrival at the clinic, her anemia had worsened, necessitating admission for blood transfusions where there was evidence of moderate schistocytosis (Figure 1) found on peripheral smear, concerning for thrombotic TTP. The patient was admitted and received plasma exchange (PLEX) for 3 days while awaiting further results. She experienced a mild urticarial reaction after the first round of PLEX, so she was pretreated with prednisone and diphenhydramine prior to the remaining two administrations. Of note, the patient’s ferritin was also abnormally elevated at > 6400 ng/mL from her baseline of 62 ng/mL at the beginning of her pregnancy. Direct antiglobulin test (DAT) demonstrated a positive polyspecific result but negative IgG and C3. Her ADAMTS13 activity returned within normal limits at 77%. Anti–double‐stranded DNA studies for Lupus testing also returned within normal limits. Flow cytometry on both her peripheral blood and bone marrow aspirate was normal. Bone marrow biopsy showed a normocellular marrow without increased blasts or reticulin fibrosis and only a slight megakaryocytic hyperplasia with left shift. Extensive evaluation for infectious (including Parvovirus, Epstein–Barr Virus, Cytomegalovirus, Human Herpesvirus‐6, and Murine Typhus), autoimmune, genetics, and acquired bone marrow failure syndromes were unrevealing. Initial lab findings from the first admission are shown in Table 2. After treatment with three rounds of PLEX therapy and pretreatment steroids, the patient’s cell counts improved from a platelet nadir of 27,000/µL, hemoglobin nadir of 7.0 g/dL, WBC nadir of 1.74 × 10^3^/µL (ANC 210/µL) to a discharge platelet count of 57,000/µL, hemoglobin of 8.0 g/dL, and WBC of 6.07 × 10^3^/µL (ANC 4530/µL). She was not discharged home with any medications at this visit.

**TABLE 2:** Lab findings during each admission.

Laboratory data	Initial admission	2 months (second admission)	14 months (last follow‐up)
Hemoglobin	7.0 g/dL	8.4 g/dL	10.7 g/dL
Hematocrit	20.3% (L)	24.2% (L)	32.3% (L)
Platelets	50,000/uL	6000/uL	403,000/uL
Sodium (Na)	131 mmol/L	133 mmol/L	141 mmol/L
Potassium (K)	2.7 mmol/L	3.2 mmol/L	4.2 mmol/L
AST	55 U/L	15 U/L	N/A
Ferritin	5181 ng/mL	N/A	N/A
INR	1.4	1.0	N/A
Urinalysis	Normal	N/A	Normal
Haptoglobin	< 10 mg/dL	82 mg/dL	N/A
Peripheral smear	Schistocytes	Spherocytes	N/A
DAT/Coombs	Negative	Positive	N/A
ADAMTS13	77.2%	N/A	N/A
Flow cytometry	Normal	N/A	N/A
Bone marrow biopsy	Normocellular with only slight megakaryocytic left shift	N/A	N/A
Parvovirus/EBV/CMV/HH6/Typhus	Negative	N/A	N/A

**FIGURE 2:**
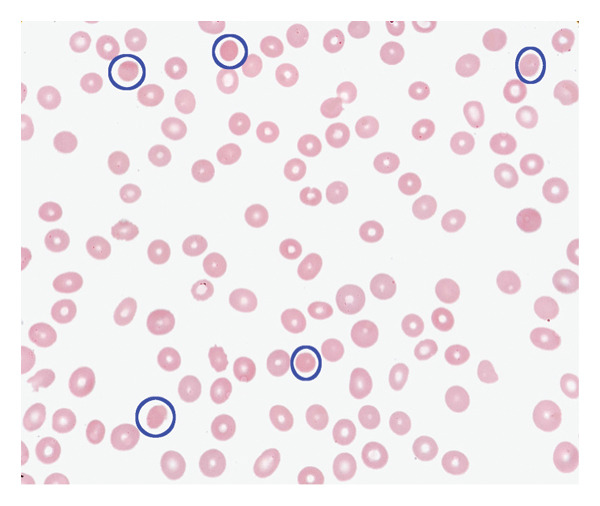
Peripheral blood smear showing spherocytes admixed with normal erythrocytes.

Two months after her initial hospitalization and 29 weeks into her pregnancy, she was readmitted for critical thrombocytopenia and anemia. In the interval between visits, another DAT had been performed which was grossly negative. However, a third DAT performed on this admission showed both positive polyspecific and IgG result, indicating the presence of IgG autoantibodies on her erythrocytes. Lab findings from the second admission are shown in Table 2. Furthermore, there was also no evidence of schistocytes on peripheral smear; however, spherocytes were now appreciated (Figure 2). Given her positive DAT along with recurrent thrombocytopenia and anemia, her presentation was most consistent with concomitant AIHA and immune thrombocytopenia, i.e., ES.

### 2.3. Follow‐Up and Outcomes

The patient was initiated on high‐dose steroids and intravenous immunoglobulin therapy (IVIG) with marked improvement in her cell counts, and she was then discharged on a steroid taper. Management of her condition was complicated by her pregnancy, and it remained unclear whether this was contributing to her disease. The patient received routine prenatal care throughout her gestation including standard scheduled obstetric checkups and fetal monitoring, and she had an otherwise uncomplicated pregnancy despite her hematological condition. She received no additional obstetric interventions but was placed on a steroid burst and taper that she was still taking at the time of her delivery. Reassuringly, the patient proceeded to have an uncomplicated delivery, and her child did not suffer from any complications during the mother’s cytopenic episodes or immediately after delivery. She finished her steroid taper 1 month after her delivery. However, her thrombocytopenia persisted, suggesting another underlying cause. Notably, she began developing diffuse, bilateral arthralgias pointing to a possible rheumatological etiology. Repeat studies including antinuclear antibody, anti–double‐stranded DNA, anti‐SSA/Ro, and anti‐Smith antibodies all returned positive confirming the diagnosis of systemic lupus erythematosus (SLE). Four months after her delivery and 8 months after her initial hospitalization, she was finally diagnosed with ES secondary to SLE and began treatment with rituximab and hydroxychloroquine. With this treatment, the patient experienced a remission of her arthralgias and normalization of her anemia and thrombocytopenia with lab values from her last follow‐up seen in Table 2.

## 3. Discussion

ES is a rare autoimmune condition characterized by two or more cytopenias, typically AIHA and immune thrombocytopenia (ITP). Population‐based data estimate an annual incidence of 1.8 per million person‐years with a prevalence of 21.3 per million persons [3]. ES is most frequently diagnosed during the fifth to sixth decades of life and is classified as either primary (idiopathic) or secondary to an underlying cause or disease state which is seen in 27%–50% of cases [5]. While it is generally associated with hematologic malignancies and SLE in adults, it can also occur with infections and primary immune deficiencies. Importantly, many of these associated conditions can independently produce cytopenias which further complicate the diagnosis and ultimately influence prognosis and management. For our patient, it was only after her delivery that SLE was identified as an underlying cause of her presentation. A list of known associations and differential diagnoses is shown in Table 3.

**TABLE 3:** Secondary associations of ES.

Etiologies for secondary Evans Syndrome	
Systemic lupus erythematosus	Common variable immunodeficiency
Primary antiphospholipid syndrome	IgA deficiency
Sjögren syndrome	B‐cell non‐Hodgkin lymphoma
Chronic myelomonocytic leukemia	Chronic lymphocytic leukemia
T‐cell non‐Hodgkin lymphoma	Autoimmune lymphoproliferative syndrome

There were a variety of clues at the patient’s initial presentation that hinted at an autoimmune pathology such as SLE as the primary cause of the patient’s cytopenias. For example, the patient’s ferritin was remarkably elevated upon admission. Ferritin is an acute phase reactant, and it is a well‐known marker for potential systemic inflammation and chronic disease [6]. While ferritin elevation is a nonspecific marker, her relative lack of bone marrow dysplasia also hinted at the underlying pathology. Aside from a slight megakaryocytic hyperplasia, her bone marrow appeared to have an insufficient response to the peripheral destruction of her red blood cells and platelets. This finding can be explained by active systemic inflammation such as SLE as this can suppress the bone marrow’s proliferative capacity [7]. Inflammatory cytokines such as IL‐6 and TNF‐α impair erythropoiesis and induce iron sequestration [6]. In effect, normocellularity represented a relative state of hypoplasia in this patient induced by an underlying inflammatory disorder. Her abnormal ferritin level together with the relative lack of hematopoiesis indicated a possible SLE flare rather than simple iron overload or some other hematologic pathology.

Furthermore, one potential reason that the patient experienced a relatively fast and robust improvement in her cell counts after her initial visit may lie in the steroids she received prior to receiving PLEX. PLEX alone would transiently remove the circulating autoantibodies and immune complexes, but it would not address the underlying immune dysregulation driving the disease. The small amount of steroids she received likely provided enough immune suppression to treat her initial flare. This would also explain why her ferritin level immediately began improving in the following days and weeks along with her cell counts.

Nonetheless, the reasons for the delay in the diagnosis of SLE as the primary diagnosis are varied. First, the lack of specificity of the elevated inflammatory markers together with the multiple treatments administered made it initially difficult to narrow the differential. An initial anti–double‐stranded DNA test was collected at the first encounter; however, it was collected 2 days after the PLEX therapy which likely reduced the circulating IgG antibodies. This may have produced the false negative given the short amount of time between the treatment and the study. Furthermore, the diagnosis of ES as the cause of the patient’s cytopenias was mainly hampered by the initial DAT results. The patient demonstrated a positive polyspecific DAT on admission prior to therapy though with negative monospecific IgG testing. This could reflect a low level of immunoglobulin below the detection threshold. Studies have shown that 5%–10% of patients with AIHA may have a negative DAT result. One reason for this is the threshold sensitivity of the test which falls around 100–500 IgG molecules per RBC. If the IgG coating on the RBC falls below the threshold, the test may fail to detect it [8]. Interestingly, the patient had a negative polyspecific result 3 weeks after her initial presentation. The reason for this discrepancy is unclear. While PLEX can reduce circulating antibodies and potentially lower the detection threshold in the short term, the bound antibodies and time between treatment and testing would make this less likely. It is also unlikely that transient immunosuppressive effects of the pretreatment steroids from her initial visit would produce these results.

Subsequent emergence of both polyspecific and IgG‐specific DAT positivity later in her course could be consistent with continued production and intensification of the warm auto–IgG antibody coating characteristic of ES. This temporal serologic progression would support an evolving autoimmune hemolytic process as would be consistent with her clinical presentation. Regardless, the follow‐up test resulting positive was vital in narrowing the diagnosis. Afterward, given the multiple associations of ES, it was not obvious whether the disease was idiopathic or secondary in our patient. The fact that she was also pregnant only confounded the investigation.

De novo SLE onset in pregnancy is uncommon but not rare. Retrospective studies from tertiary centers have found this occurrence in up to 30% of all SLE pregnancies [9]. Still, pregnancy is known to trigger SLE activity, specifically manifesting as renal or hematological symptoms in most cases [10]. Therefore, it is also likely that her development of ES was a presentation of a severe flare of previously undiagnosed, subclinical SLE. It was only after our patient delivered and afterward began experiencing intraarticular manifestations that SLE was suspected as the primary diagnosis confirmed with serological testing.

Management of primary ES has historically been extrapolated from treatment of ITP and AIHA given its rarity and unclear pathogenesis [2]. First‐line treatment involves treating both the ITP and AIHA with corticosteroids based on current guidelines [11, 12]. Retrospective studies suggest overall response rates of roughly 80% of patients presenting with both AIHA and ITP with a complete response rate between 50% and 67% of responders [4]. Although data on remission rates for corticosteroid monotherapy are limited for ES, studies have found 1‐year remission rates of 20%–30% for isolated ITP and 33% for AIHA [2].

IVIG is recommended for patients with low platelet counts and can be considered first‐line if steroids are contraindicated or ineffective [13]. In ES patients whose ITP was treated with IVIG, response rates were as high as 60%. However, in cases of isolated AIHA, patients achieved only a partial response (Hb level of at least 10 g/dL) [4, 14]. Transfusion support is necessary in symptomatic anemia. However, transfusing platelets is not recommended for cases of isolated ITP in the absence of life‐threatening bleeds due to insufficient evidence that transfusing platelets improve outcomes [15].

Second‐line treatments for ES are reserved for patients with inadequate responses to prior interventions and typically involve the use of rituximab for long‐term immunosuppression, and/or splenectomy to reduce immune‐mediated erythrocyte and platelet sequestration and destruction. Rituximab’s efficacy has been demonstrated in treating both isolated AIHA and isolated ITP. The RAIHA study, which was a randomized and double‐blinded trial involving patients with isolated AIHA treated with rituximab, demonstrated a 75% overall response (complete and partial response) at 1 year with a relapse‐free survival of over 80% at 3 years [16]. Furthermore, for isolated ITP, the French prospective ITP registry found an initial response of 60.9% (including a CR of 32.3%) with relapse‐free survival of 29.4% at 5 years with rituximab [17]. In ES, the initial response rate in both patients’ anemia and thrombocytopenia to rituximab was found to be 82% which fell to 64% at 1 year [4]. In pregnant patients with primary ES, corticosteroids are considered frontline therapy and other modalities such as rituximab and are not recommended [2].

For secondary ES, the treatment approach varies based on the primary disorder and should also address the underlying ES. A retrospective cohort study found that patients with ES secondary to SLE treated with rituximab achieved an overall response in 60% of cases with a complete response in 50% of cases [18]. This was evident in the management of our patient’s SLE after delivery with her successful response to rituximab treatment.

ES in pregnancy is a very rare disorder with a few published cases. A systematic review conducted by the European Journal of Obstetrics and Gynecology and Reproductive Biology Group identified only 14 cases in the medical literature of ES occurring during pregnancy and reviewed 11 of the pregnancies with a median age at diagnosis of 21 years [19]. In around 40% of cases, the diagnosis of ES was made prior to pregnancy, but when diagnosed during pregnancy, the diagnosis was made between the 14th and 38th weeks of gestation. AIHA occurred simultaneously with thrombocytopenia in all but two of the cases, and none of the women had neutropenia. Only 2 of the babies were stillborn, one to a mother with known syphilis and found to have an intracranial subdural hematoma at 35 weeks gestation, and the other delivered at 32 weeks gestation with evidence of autoimmune hemolysis from the mother’s ES. Only one baby was found to have autoimmune hemolysis postpartum that resolved spontaneously without treatment. All the women received prednisolone, however in only 4 of the pregnancies were steroids, the only therapy with an effective response. Other treatments that demonstrated efficacy include IV gamma globulin and splenectomy. However anti‐D globulin did not produce a response. One mother received no benefit from any of the above treatments or rituximab and required weekly PLEX for 1 month until remission. The women were followed postpartum from 2 months to 8 years after delivery without evidence of hemolysis or thrombocytopenia and most without treatment [14]. The patient’s pregnancy contributed to the complexity of diagnosis and treatment. Recognizing ES and its related conditions is essential for identifying the underlying cause and choosing the best treatment strategy.

Outcomes for patients with ES are generally poorer than that in isolated autoimmune cytopenias with reported median survival for ES of approximately 7 years from diagnosis. Roughly one‐third of all deaths occur within the first year of diagnosis [3]. Mortality is most commonly related to hemorrhage or secondary to an underlying hematologic neoplasm. The severity of anemia at diagnosis has been associated with worse outcomes including increased relapse rates and overall survival. Interestingly, relapses increase incrementally with declining hemoglobin levels [20]. When stratified by etiology, patients with secondary ES have inferior survival rates with a reported median of around 1.7 years compared to nearly 11 years for primary disease. Five‐year survival rates continue the same trend at 38% for secondary ES vs. 75% for primary. Notably, median survival for both primary and secondary ES is lower than isolated autoimmune cytopenias such as AIHA and ITP with reported median survival of 8.7 and 12.7 years, respectively [3]. This suggested that the increased mortality in ES may be attributed to the presence of other concurrent disease processes such as malignancies or immunodeficiencies.

### 3.1. Learning Points

This case report describes a novel case of ES occurring in a 21‐year‐old gravid patient. This already rare disease is even more uncommon among pregnant women, necessitating a thorough hematologic workup. Identification of possible secondary causes is paramount given the high rates of mortality and need for directed therapies to induce a remission. The wide variety of diseases that can produce ES require their own workup and can range from rheumatologic disease to cancer. Recognizing and understanding these associations is crucial for timely diagnosis and effective management, ultimately improving patient outcomes.

## Author Contributions

All authors contributed to the literature review, writing of original draft, and editing of original draft.

Christian Bittner, MD, Niels A. Ryden, MD, Charles Jacocks, MD, and Chung‐Ting J. Kou, DO, had full access to all of the data in this study and take complete responsibility for the integrity of the data and the accuracy of the data analysis.

## Funding

No funding was received for this manuscript.

## Disclosure

The opinions and assertions expressed herein are those of the author(s) and do not necessarily reflect the official policy or position of the Uniformed Services University of the Health Sciences or the Department of Defense. All authors have read and approved the final version of the manuscript.

## Consent

Informed consent for the publication from the patient was obtained.

## Conflicts of Interest

The authors declare no conflicts of interest.

## Data Availability

Data and material are available from the corresponding author on reasonable request.
